# Genetic analysis of the relation of telomere length‐related gene (*RTEL1*) and coronary heart disease risk

**DOI:** 10.1002/mgg3.550

**Published:** 2019-01-08

**Authors:** Shijuan Lu, Jianghua Zhong, Miao Wu, Kang Huang, Yilei Zhou, Zanrui Zhong, Qiang Li, Honghao Zhou

**Affiliations:** ^1^ Department of Clinical Pharmacology, Xiangya Hospital Central South University Changsha China; ^2^ Institute of Clinical Pharmacology Hunan Key Laboratory of Pharmacogenetics Central South University Changsha China; ^3^ Department of Cardiology, Haikou People’s Hospital Central South University Xiangya School of Medicine Affliated Haikou Hospital Haikou China

**Keywords:** case–control study, coronary heart disease, genetic polymorphism, *RTEL1*, SNP

## Abstract

**Background:**

Regulator of telomere elongation helicase 1 (*RTEL1*), a telomere length‐related gene, is closely linked to cancer and age‐related diseases. The aim of this study was to investigate the association between genetic polymorphisms in the *RTEL1* gene and coronary heart disease (CHD) risk.

**Methods:**

In this case–control study, which includes samples from 596 CHD patients and 603 healthy controls, five SNPs in *RTEL1* were selected. The genotypes were studied using the Agena MassARRAY platform, and the statistical analyses were performed using the chi‐square and Fisher's exact tests, genetic model analysis, and haplotype analysis.

**Results:**

In the allele model, using the chi‐square test, we found that the patients with the “G” allele of rs6010620 and the “C” allele of rs4809324 in the *RTEL1* gene showed a decreased risk of CHD once the results were adjusted for age and gender. In the genetic model, logistic regression analyses revealed that the rs6010620 polymorphism conferred a decreased risk of CHD in the codominant model (OR = 0.52, 95% CI: 0.31–0.88, *p* = 0.007 for the “G/G” genotype) and the recessive model (OR = 0.49, 95% CI: 0.30–0.80, *p = *0.004 for the “G/G” genotype). In addition, the haplotype “G_rs6010620_T_rs6010621_T_rs4809324_” of *RTEL1* was associated with a 0.03‐fold decreased risk of CHD once the results were adjusted for age and gender (OR = 0.03, 95% CI: 0.01–0.12, *p* < 0.001).

**Conclusion:**

Our findings have demonstrated that the genetic variants of *RTEL1 *may have a protective role against CHD risk.

## 1 INTRODUCTION

Coronary heart disease (CHD) is one of the most common cardiovascular diseases, and one of the most common causes of death and disability globally (Sacks et al., 2017). The pathophysiological mechanism of coronary heart disease is atherosclerosis, which is characterized by the deposition of excessive cholesterol in the arterial intima (Lusk et al., 2014). The majority of CHD cases can be explained by the interaction of genetic and environmental factors (Peyser, 1997). Because CHD is an age‐related disease, however, interindividual variation in risk of CHD might result from variation in the rate of biological aging (Coronary Artery Disease, 1938).

Telomeres are protein‐bound DNA repeat structures that are located at the extreme ends of chromosomal DNA where they play an important role in maintaining genomic stability (Rhyu, 1995). They are also a marker of biological aging (Blasco, 2005). In previous studies, mean leukocyte telomere length (LTL) was found to be a predictor of CHD events in middle‐age (Spyridopoulos et al., 2009). A case–control study confirmed the association between shorter LTL and an increased risk of coronary artery disease in European populations, which supports the hypothesis that differences in biological aging might contribute to the differences in disease risk and age of CHD onset (Li et al., 2017). In recent years, more studies have revealed that several genes and gene variants are strongly associated with CHD, including *TN1P1*, *MPHOSPH6* and *ZNF208 *(Song et al., 2017), *SH2B3* and *SMARCA4 *(Ji et al., 2017), *TERT *(Han et al., 2017), and *APOB *(Al‐Bustan, Ismael, Al‐Serri, & Al‐Rashdan, 2017).

*RTEL1* (OMIM: 608833) is a DNA helicase that plays important roles in setting telomere length, maintaining telomeres, and repairing DNA in mice (Barber et al., 2008). Recently, genome‐wide association studies (GWAS) have shown that *RTEL1* dysfunction appears to be closely related to certain cancers and age‐related diseases such as lung cancer (Yan et al., 2016), glioma (Du et al., 2014), astrocytoma (Jin et al., 2015, 2013), stroke (Cai et al., 2017a), and colorectal cancer (Li et al., 2016). However, few studies have investigated the association between genetic variants in *RTEL1* and the risk of CHD. We performed a case–control study to analyze the association between six single nucleotide polymorphisms (SNPs) in *RTEL1 *and the risk of CHD in a Chinese Han population.

## 2 MATERIALS AND METHODS

### 2.1 Ethics statement

The study protocol was approved by the ethics committee of Haikou People's Hospital. Written informed consent was obtained from all participants after a full explanation of the study. All samples were coded to protect donor identity. The experimental protocol was implemented in accordance with the approved guidelines.

### 2.2 Study subjects

This study included 596 CHD patients and 603 healthy controls. All subjects in our study were recruited from Haikou People's Hospital, Hainan, China. Patients were unrelated subjects ages 19–83 years old. The controls were recruited from routine healthy examinations in the same hospital. Patients were diagnosed with CHD using standard coronary angiography. Coronary angiography had to reveal 50% narrowing of the lumina of at least one of the major coronary arteries for a patient to be included in the study. Subjects with myocardial infarction, stable angina, and unstable angina were classified as CHD subjects. Non‐CHD controls have no congenital heart disease, familial hypercholesterolemia, end‐stage renal disease, or known vasculitides.

### 2.3 SNP genotyping

Five SNPs in *RTEL1 *with minor allele frequencies (MAF) of >0.05 were identified in an association analysis of a Beijing Chinese population. The selected SNPs were reported to be associated with CHD and other cardiovascular disease risk. The SNP was found within an intronic region and was unlikely to possess any functional significance, according to the RegulomeDB. The GTEx result for this SNP shows that it is not known to be associated with gene expression in the most relevant tissue (vascular or peripheral nerve); however, the SNP and the associated variants in LD are known as eQTLs in artery tissue (Supporting information Table S2).

A GoldMag‐Mini Purification Kit (GoldMag Co. Ltd. Xian City, China) was used to extract genomic DNA from whole‐blood samples. DNA samples were stored at −80°C prior to analysis. DNA concentrations were measured using a NanoDrop 2000 (Thermo Scientific, Waltham, Massachusetts, USA). Agena MassARRAY Assay Design 4.0 software was used to design a multiplexed SNP MassEXTEND assay, and SNP genotyping was performed using the Agena MassARRAY RS1000 with manufacturer protocols. The PCR primers for each SNP are shown in Supporting information Table S1. Agena Typer 4.0 software was used to perform data management and analyses.

### 2.4 Statistical analysis

All statistical analyses were performed using SPSS 19.0 software for Windows (SPSS, Chicago, IL). Allele and genotype frequencies were determined using direct counts. Hardy–Weinberg equilibrium values for each SNP were determined using an exact test to compare the expected frequencies of genotypes in controls. Allele and genotype frequencies in CHD patients and controls were calculated using chi‐squared and Fisher's exact tests. Associations between SNPs and the risk of steroid‐induced CHD were tested in genetic models using PLINK software (Version 1.07). Odds ratios (ORs) and 95% confidence intervals (CIs) were calculated using unconditional logistic regression analysis with adjustment for gender and age (Bland & Altman, 2000). Finally, the Haploview software package (version 4.2) and SHEsis software platform (http://www.nhgg.org/analysis/) were used to estimate pairwise linkage disequilibrium (LD), haplotype construction, and genetic association at polymorphism loci (Barrett, Fry, Maller, & Daly, 2005; Shi & He, 2005). All *p* values were two‐sided, and *p* ≤ 0.05 was considered statistically significant.

## 3 RESULTS

### 3.1 Characteristics of the participants

A total of 596 CHD cases (376 men and 220 women; mean age, 61.44 ± 11.16 years) and 603 controls (469 men and 134 women; mean age, 48.24 ± 13.05 years) were included in the study. The clinical characteristics of the cases and controls are shown in Table 1. There were no significant differences in the age and gender distributions between the case and control groups (*p* < 0.05).

**Table 1:** Basic characteristics

Parameters	Case	Control	*p* value
No	596	603	<0.001
Males	376 (63.1%)	469 (77.8%)	
Females	220 (36.9%)	134 (22.2%)	
Mean age	61.44 ± 11.16	48.24 ± 13.05	<0.001
ALT (U/L)	31.17 ± 2.13		
AST (U/L)	36.62 ± 2.15		
GGT (U/L)	44.59 ± 3.82		
TP (g/L)	66.43 ± 0.31		
GLU (mmol/L)	6.35 ± 0.11		
TG (mmol/L)	1.80 ± 0.06		
TC (mmol/L)	4.09 ± 0.07		
HDL‐C (mmol/L)	1.14 ± 0.01		
LDL‐C (mmol/L)	1.93 ± 0.03		
APOA1 (g/L)	1.27 ± 0.01		
APOB (g/L)	1.01 ± 0.02		
Lp(a)(mg/L)	240.1 ± 12.11		
PLT (109/L)	169.47 ± 3.55		
PCT (%)	1.14 ± 0.15		
MPV (fl)	13.12 ± 0.32		
PDW (%)	14.22 ± 0.16		

Note

*p* < 0.05 indicates statistical significance.

ALT: alanine aminotransferase; apoA: apolipoprotein A; APOB: apolipoprotein B; AST: aspartate aminotransferase; GGT: gamma‐glutamyl transpeptidase; GLU: glucose; HDL: high‐density lipoprotein; LDL: low‐density lipoprotein; LP(a): lipoprotein; MPV: Mean Platelet Volume; PCT: plateletcrit; PDW: platelet distribution width; PLT: platelet; TC: total cholesterol; TG: triglyceride; TP: total protein.

### 3.2 Associations between RTEL1 SNPs and CHD risk

Five SNPs within the *RTEL1* locus were genotyped in CHD patients and healthy controls (Table 2). Using chi‐square tests, we determined that rs6010620 and rs4809324 were associated with a decreased risk of CHD (rs6010620: OR = 0.78, 95% CI = 0.65–0.93, *p* = 0.005; rs4809324: OR = 0.08, 95% CI = 0.04–0.16, *p* = 2.74E ‐ 21).

**Table 2:** Allele frequencies in cases and controls and odds ratio

SNP	Chromosome	Position	Allele	MAF		HWE *p*	OR (95% CI)	*p* a	*p* b
				Case	Control				
rs6089953	20	62291008	G/A	0.261	0.280	0.840	0.91（0.76–1.09）	0.297	0.0594
rs6010620	20	62309839	G/A	0.265	0.317	0.500	0.77（0.65–0.93）	0.005^*^	0.001^*^
rs6010621	20	62310872	G/T	0.263	0.271	0.837	0.96（0.80–1.15）	0.667	0.133
rs4809324	20	62318220	C/T	0.098	0.107	0.526	0.08（0.04–0.16）	2.7E‐21^*^	5.4E‐22^*^
rs2297441	20	62327582	A/G	0.317	0.325	0.517	0.96（0.81–1.14）	0.677	0.133

Note

95% CI: 95% confidence interval; HWE: Hardy–Weinberg equilibrium; OR: odds ratio.

Bold highlights the value of P and OR(95%CI) with statistical significance.

^*^p ^a^ **< 0.05 indicates statistical significance.

^*^p ^b^ < 0.01 indicates statistical significance.

a *p *values were calculated from a chi‐square test or Fisher's exact test.

b *p* values were adjusted by Bonferroni correction.

### 3.3 Associations between genotype frequencies and CHD risk

As is shown in Table 3, logistic regression analyses revealed that the rs6010620 polymorphism in the *RTEL1* gene conferred a decreased risk of CHD in the codominant model (adjusted: OR = 0.52, 95% CI: 0.31–0.88, *p* = 0.007 for the “G/G” genotype) and the recessive model (adjusted: OR = 0.49, 95% CI: 0.30–0.80, *p = *0.004 for the “G/G” genotype).

**Table 3:** Genotypic model analysis of the relationship between SNPs and coronary heart disease risk

SNPs	Model	Genotype	control	case	OR (95% CI)	*p* a‐value	*p* b‐value
Rs6089953	Codominant	A/A	311 (51.6%)	317 (53.3%)	1	0.71	0.142
		A/G	246 (40.8%)	245 (41.2%)	1.02 (0.78–1.33)		
		G/G	46 (7.6%)	33 (5.5%)	0.81 (0.47–1.39)		
	Dominant	A/A	311 (51.6%)	317 (53.3%)	1	0.91	0.182
		A/G‐G/G	292 (48.4%)	278 (46.7%)	0.98 (0.76–1.28)		
	Recessive	A/A‐A/G	557 (92.4%)	562 (94.5%)	1	0.41	0.082
		G/G	46 (7.6%)	33 (5.5%)	0.80 (0.47–1.36)		
	Log‐additive	‐‐‐	‐‐‐	‐‐‐	0.96 (0.77–1.18)	0.67	0.134
Rs6010620	Codominant	A/A	270 (47.2%)	315 (52.9%)	1	0.007*	0.0014^*^
		A/G	241 (42.1%)	246 (41.3%)	1.19 (0.90–1.59)		
		G/G	61 (10.7%)	35 (5.9%)	**0.52 (0.31–0.88)**		
	Dominant	A/A	270 (47.2%)	315 (52.9%)	1	0.79	0.158
		A/G‐G/G	302 (52.8%)	281 (47.1%)	1.04 (0.79–1.36)		
	Recessive	A/A‐A/G	511 (89.3%)	561 (94.1%)	1	0.004*	0.0008^*^
		G/G	61 (10.7%)	35 (5.9%)	**0.49 (0.30–0.80)**		
	Log‐additive	‐‐‐	‐‐‐	‐‐‐	0.90 (0.73–1.10)	0.31	0.062
Rs6010621	Codominant	T/T	318 (52.9%)	317 (53.2%)	1	0.76	0.152
		G/T	240 (39.9%)	244 (40.9%)	1.10 (0.84–1.45)		
		G/G	43 (7.2%)	35 (5.9%)	0.98 (0.57–1.69)		
	Dominant	T/T	318 (52.9%)	317 (53.2%)	1	0.54	0.108
		G/T‐G/G	283 (47.1%)	279 (46.8%)	1.09 (0.84–1.41)		
	Recessive	T/T‐G/T	558 (92.8%)	561 (94.1%)	1	0.83	0.166
		G/G	43 (7.2%)	35 (5.9%)	0.94 (0.56–1.60)		
	Log‐additive	‐‐‐	‐‐‐	‐‐‐	1.05 (0.85–1.29)	0.68	0.136
Rs4809324	Codominant	T/T	479 (79.4%)	457 (81.2%)	1	0.92	0.184
		T/C	119 (19.7%)	102 (18.1%)	0.96 (0.69–1.35)		
		C/C	5 (0.8%)	4 (0.7%)	1.26 (0.30–5.25)		
	Dominant	T/T	479 (79.4%)	457 (81.2%)	1	0.88	0.176
		T/C‐C/C	124 (20.6%)	106 (18.8%)	0.98 (0.70–1.36)		
	Recessive	T/T‐T/C	598 (99.2%)	559 (99.3%)	1	0.74	0.148
		C/C	5 (0.8%)	4 (0.7%)	1.27 (0.31–5.28)		
	Log‐additive	‐‐‐	‐‐‐	‐‐‐	0.99 (0.72–1.35)	0.95	0.190
Rs2297441	Codominant	G/G	271 (44.9%)	276 (46.3%)	1	0.86	0.172
		A/G	272 (45.1%)	262 (44%)	0.96 (0.73–1.26)		
		A/A	60 (9.9%)	58 (9.7%)	1.09 (0.69–1.72)		
	Dominant	G/G	271 (44.9%)	276 (46.3%)	1	0.88	0.176
		A/G‐A/A	332 (55.1%)	320 (53.7%)	0.98 (0.76–1.27)		
	Recessive	G/G‐A/G	543 (90%)	538 (90.3%)	1	0.64	0.128
		A/A	60 (9.9%)	58 (9.7%)	1.11 (0.71–1.73)		
	Log‐additive	‐‐‐	‐‐‐	‐‐‐	1.01 (0.83–1.23)	0.93	0.186

Note

95% CI: 95% confidence interval; OR: odds ratio; SNP: single nucleotide polymorphism.

Bold highlights the value of P and OR(95%CI) with statistical significance.

*p ﹤ 0.05 indicates statistical significance.

^*^p ^b^ **﹤ 0.01 indicates statistical significance.

a *p* values were calculated by unconditional logistic regression analysis with adjustments for age and gender.

b *p* values were adjusted by Bonferroni correction.

### 3.4 Associations between haplotype analyses and CHD risk

**Figure 1:**
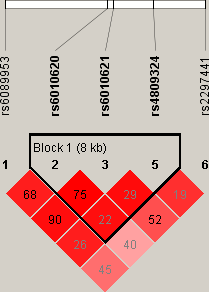
Linkage disequilibrium (LD) plots containing five SNPs from *RTEL1*

Linkage disequilibrium and haplotype analyses of the SNPs in the case and control samples were further studied. Haplotype analysis revealed the block in the *RTEL1* genes. *RTEL1* genes rs6089953, rs6010620, and rs6010621 had very strong linkage disequilibria (Figure 1); compared to the “ATT” wild‐type, the “GTT” haplotype was associated with a decreased risk of CHD (OR = 0.03, 95% CI = 0.01–0.12, *p* < 0.0001) (Table 4).

**Table 4:** *RTEL1* haplotype frequencies and the association with coronary heart disease risk

	rs6010620	rs6010621	rs4809324	Case	Control	Freq	Without adjustment		With adjustment	
							OR (95% CI)	*p* a	OR (95% CI)	*p* b
1	A	T	T	0.735	0.67	0.7016	1	‐‐‐	1	‐‐‐
2	G	G	T	0.166	0.168	0.1672	0.89 (0.71–1.12)	0.32	1.03 (0.80–1.33)	0.82
3	G	G	C	0.0.97	0.105	0.1013	0.82 (0.62–1.09)	0.17	0.94 (0.68–1.29)	0.69
4	G	T	T	0.002	0.054	0.029	0.03 (0.01–0.11)	<0.001^*^	0.03 (0.01–0.12)	<0.001^*^

Note

95% CI: 95% confidence interval; OR: odds ratio.

a *p *values were calculated from unconditional logistic regression analysis.

b *p* values were calculated by unconditional logistic regression analysis with adjustments for age and gender.

*p ≤ 0.05 indicates statistical significance.

## 4 DISCUSSION

**Figure 2:**
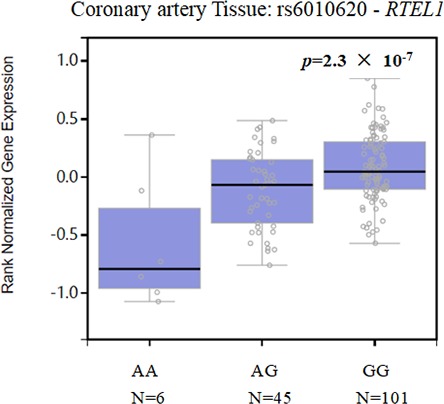
*RTEL1* (rs6010620) expression in coronary artery tissues

The present case–control study of 596 CHD patients and 603 healthy controls was designed to investigate the associations between five SNPs in the *RTEL1* gene and the risk of CHD in a Chinese Han population. We found that rs6010620 and rs4809324 were associated with a decreased risk of CHD. In addition, the “G_rs6010620_T_rs6010621_T_rs4809324_” *RTEL1* haplotype was associated with a decreased risk of CHD. Our findings have shed new light on the *RTEL1 *polymorphisms that may contribute to the protection of CHD. Additionally, eQTL analysis based on the GTEx database (https://gtexportal.org/home/) indicated that the rs6010620 polymorphisms may alter the expression level of *RTEL1 *in coronary artery tissues (Figure 2).

Regulator of Telomere Elongation Helicase 1 (*RTEL1*), an essential DNA helicase, is located in 20q13.3 and includes 40 exons. *RTEL1* disassembles a variety of DNA secondary structures to facilitate DNA replication, repair, and recombination processes, thereby helping to maintain telomere integrity (Vannier, Sarek, & Boulton, 2014). Several studies have shown that a substantial proportion of the marked interindividual variation in mean telomere length is genetically determined (Slagboom, Droog, & Boomsma, 1994). In combination, this suggests that individuals who have inherited and shorter telomeres might be more prone to coronary heart disease. It is thus possible that the association of shorter telomeres with an increased risk of coronary heart disease may have a genetic basis (Codd et al., 2013). Any genetic susceptibility could be exacerbated or retarded by postnatal effects on telomere length. If true, this observation could not only partially explain the genetic basis of coronary heart disease, but also its variable age of onset.

Furthermore, previous studies have revealed overexpression of the *RTEL1* genomic locus in several cancers such as breast, lung, esophagus, gastric, and colorectal cancer (Muleris, Almeida, Gerbault‐Seureau, Malfoy, & Dutrillaux, 1995). Additionally, a mouse model study revealed that *RTEL1* could support cell growth by participating in Wnt/β‐catenin signaling, which suggests that *RTEL1* may be considered to be an oncogene (Wu, Sandhu, Nabi, & Hao, 2012). However, in the past decade, *RTEL1* variants have been associated with the decreased risk of several brain cancers and age‐related disease including glioma, astrocytoma, glioblastomas, and congenital dyskeratosis. In our study, we investigated six SNPs in *RTEL1*: rs6089953, rs6010620, rs6010621, rs2297440, rs4809324, and rs2297441. Among these SNPs, Ding Y et al., (Ding et al., 2017) reported the presence of rs4809324 was associated with increasing the COPD risk. The presence of rs2297441 was found to be associated with Crohn's disease in Canadian children (Amre et al., 2009). The presence of rs6010620 was found to increase the risk of glioma (Zhao, Bian, Zhu, Zou, & Tang, 2014). Cai et al. (2017b) reported the associations between single nucleotide polymorphisms in the *RTEL1* gene and stroke risk, and the result showed that the rs6010620, rs6010621, and rs6089953 were associated with an increased risk of stroke. However, Olivier, Charbonnel, Amiard, White, and Gallego (2018) showed *RAD51* and *RTEL1* gene could compensate telomere loss and protect cell stability when telomere was absent. And another study indicated that the presence of rs6010620 and rs2297440 resulted in a decreased risk of astrocytoma (Jin et al., 2013). Rong et al. (2017) found rs6089953, rs6010621, and rs2297441 were also associated with a decreased risk of HAPE. In our study, we found that the presence of rs6010620 and rs4809324 was associated with a decreased risk of CHD. This is consistent with previous research results. As far as we know, we are the first to report the association between the *RETL1* polymorphisms rs6010620 and rs4809324, and CHD risk. More studies should investigate these SNPs using more clinical data with bigger samples. This result may provide a new data to facilitate earlier diagnosis and promote early prevention, and shed light on the new candidate genes and new ideas for the study of subsequent occurrence mechanism of CHD. However, some potential limitations in our current study should be considered when analyzing the results. Our study only conducted preliminary basic research. Moreover, further functional studies and larger population‐based prospective studies are required to fully understand the genetic factors underlying CHD.

## 5 CONCLUSION

Our results indicate that the rs6010620 and the rs4809324 polymorphisms in *RTEL1* are associated with CHD in a Chinese Han population. These SNPs may serve as prognostic biomarkers for CHD in the Chinese Han population.

## CONFLICTS OF INTEREST

The authors have no conflicts of interest to report.
