# Supplementary material for: Genetic analysis of the relation of telomere length‐related gene (RTEL1) and coronary heart disease risk
Source: Mol Genet Genomic Med. 2019 Jan 8;7(3):e550. doi: 10.1002/mgg3.550 (PMC6418357; doi:10.1002/mgg3.550)
Supplement: Supplementary file 1 [file MGG3-7-na-s001.docx]

**Supplymentary table S1**: Primers used for this study

| SNP ID | First PCR primer | Second PCR primer | UEP SEQ |
| --- | --- | --- | --- |
| rs6089953 | ACGTTGGATGCCCTTCAAAGGACGA TCGTT | ACGTTGGATGGCGTCTGTCATAAAAAGGGC | GGGTTCCAGGTGGGGTC |
| rs6010620 | ACGTTGGATGGCCTGTTTTCCCTTTTTGAG | ACGTTGGATGCTCTCAACATCTCAGCAACC | CAGATCATGCAAAGCAGG |
| rs6010621 | ACGTTGGATGACCCCATCCCTCCCCTCTGA | ACGTTGGATGAGCACGAGAACAGCACCGAG | ACAGCACCGAGGAAAAG |
| rs4809324 | ACGTTGGATGAGCCGGTGCACAGATTCCAA | ACGTTGGATGGAGAAGTCAAGTGACATCAG | GTCAGAGGTCAATGGAACA |
| rs2297441 | ACGTTGGATGCTCAACTCCCACCACCAAG | ACGTTGGATGGTGTCCACTTTTAA TCAGGG | GACAGGGCTCTCTAATAAA |

**Supplymentary table S2:** GTEx results for this SNP in *RTEL1* gene expression in the most relevant tissue.

| SNP | Gene | Effect | *p*-value | Tissue |
| --- | --- | --- | --- | --- |
| rs6089953 | *RTEL1* | 0.4 | 6.60E-23 | Artery - Tibial |
| rs6089953 | *RTEL1* | 0.35 | 3.00E-13 | Artery - Aorta |
| rs6089953 | *RTEL1* | 0.3 | 6.80E-07 | Artery - Coronary |
| rs6010620 | *RTEL1* | 0.41 | 3.50E-23 | Artery - Tibial |
| rs6010620 | *RTEL1* | 0.36 | 9.50E-14 | Artery - Aorta |
| rs6010620 | *RTEL1* | 0.32 | 2.30E-07 | Artery - Coronary |
| rs6010621 | *RTEL1* | 0.4 | 4.10E-23 | Artery - Tibial |
| rs6010621 | *RTEL1* | 0.36 | 1.60E-13 | Artery - Aorta |
| rs6010621 | *RTEL1* | 0.32 | 2.30E-07 | Artery - Coronary |
| rs2297441 | *RTEL1* | 0.33 | 8.50E-16 | Artery - Tibial |
| rs2297441 | *RTEL1* | 0.31 | 3.50E-11 | Artery - Aorta |
| rs2297441 | *RTEL1* | 0.28 | 1.7E-06 | Artery - Coronary |
